# Correlation of body compositions and bone mineral density in postmenopausal women with different body mass index

**DOI:** 10.3389/fendo.2025.1642801

**Published:** 2025-11-25

**Authors:** Song Ge, Min Li, Xiaoxue Bao, Gege Wu, Mengcong Liu, Wei Zhang, Yukun Li, Yan Wang

**Affiliations:** 1 Department of Endocrinology, Hebei Medical University Third Hospital, Shijiazhuang, Hebei, China; 2 Department of Medical Imaging, Hebei Medical University Third Hospital, Shijiazhuang, Hebei, China

**Keywords:** postmenopausal women, body composition, body mass index, bone mineral density, lean mass, fat mass

## Abstract

**Background:**

The association between obesity and bone mineral density (BMD) is controversial. Body composition parameters have been found to be strongly correlated with BMD. Body mass index(BMI)cannot distinguish between muscle and adipose tissue. The objective of this study was to evaluate the association of body composition with BMD in postmenopausal women with different BMI.

**Methods:**

356 postmenopausal women were divided into three groups(normal weight, overweight and obesity)according to BMI. BMD and body composition components were obtained by Dual-energy X-ray Absorptiometry. The collected data served as the training set for model development, while datasets from the NHANES database were utilized as a validation set to assess model performance. Multivariable linear regression models evaluated associations between parameters of body composition and BMD in different BMI participants.

**Results:**

In univariate analysis, BMI, total fat mass, soft lean mass (SLM), appendicular skeletal muscle mass, relative skeletal muscle index (RSMI) were positively correlated with BMD at all sites (r = 0.181–0.388, all *P* < 0.01).In normal weight subjects, multivariate regression models consistently revealed positive associations of SLM and android-to-gynoid ratio (AOI) with BMD across lumbar spine, hip, and femoral neck sites (Model 1: SLM Sβ=0. 260-0. 313, all P<0.001; AOI Sβ=0.224-0. 289, all P<0.05. Model 2: RSMI Sβ=0.182-0.218, all P<0.01; AOI Sβ=0.174-0.235, all P<0.05). Among overweight subjects, AOI showed site-specific correlations with lumbar spine BMD in both models (Model 1 Sβ=0.207; Model 2 Sβ=0.193), while SLM maintained positive associations with all sites (Sβ=0.238-0.246, P<0.01) and RSMI with femoral neck BMD (Sβ=0.196, P<0.05). No significant body composition-BMD correlations were observed in obese subjects. External validation with NHANES database confirmed model robustness, with all significant β coefficients from the validation set falling within the training set’s 95% CIs.

**Conclusions:**

The study demonstrates that the effect of body compositions on BMD varies by BMI classification and site-specific differences in postmenopausal women. Increased abdominal fat may confer a potential benefit for BMD in non-obese women with relative metabolic health. Conversely, optimizing body composition by reducing body fat and increasing muscle mass remains crucial for skeletal health in postmenopausal women.

## Introduction

Osteoporosis (OP) is the most common metabolic bone disease in postmenopausal women. The prevalence of OP in people aged 40 years or older was approximately 20.6% in women (1). Bone mineral density (BMD) is an essential marker for assessing and identifying OP. Osteoporosis is characterized by low bone mass, increased bone fragility, and increased susceptibility to fracture, which is associated with substantial morbidity, mortality, and economic costs. The risk of morbidity is increased following an osteoporotic fracture, including an 86% risk of subsequent fractures, particularly in the first two years, and an overall risk of death of 20% in the first year after a hip fracture. These risks increase significantly with the age at the time of fracture (2). Meta-analysis study has indicated that obesity is positively associated with BMD and negatively correlated with OP (3). However, recent study showed that obesity may have a negative effect on BMD (4). Thus, the association between obesity and BMD is controversial and has not been fully explained.

Body mass index(BMI)is the epidemiological and clinical parameter used to define obesity in most of the studies. Nevertheless, BMI is unable to accurately predict abdominal obesity, and cannot distinguish between muscle and adipose tissue. Moreover, there are differences in the relationships of BMI to body composition (5). Studies have shown that BMD is strongly associated with some parameters of body composition. Examples of such parameters include lean mass (LM), fat mass (FM), android-to-gynoid fat ratio (AOI) (6, 7). LM, also known as fat-free body mass, is the weight of the body’s components other than fat and consists of the weight of the body’s cells, extracellular water and the fat-free solid fraction (bone and muscle). The AOI is a valuable indicator of central fat accumulation that correlates with BMD. Similarly, there are many disagreements in the literature about the effect of FM, LM or AOI on BMD. Previous reports have found that both FM and LM equally contribute to increase in bone mass among postmenopausal women (8, 9). Both LM and FM were positively correlated with total and regional BMD. In contrast, some studies suggested that FM and AOI were negatively associated with BMD (10, 11). For instance, one investigation among Americans aged 20–59 reported an inverse relationship between adipose tissue levels across various anatomical regions and BMD. In contrast, other studies have asserted a positive association between AOI and BMD (12).

The relationship between body composition and BMD in postmenopausal women appears complex and warrants further investigation. While body composition’s influence on BMD is recognized, its specific role across different BMI categories is not well-characterized. Moreover, few studies have comprehensively examined how body composition components relate to BMD at various skeletal sites among postmenopausal women stratified by BMI. To address this gap, this study aimed to investigate the associations between body composition components and site-specific BMD, stratified by BMI category, in normal-weight, overweight, and obese postmenopausal women.

## Methods

### Study subjects

Present study was a cross-sectional survey. A total of 356 postmenopausal women were enrolled in the study from November 2021 to October 2025 at the Third Hospital of Hebei Medical University. All participants underwent BMD measurement and body compositions assessment. The criteria for postmenopausal women were natural menopause for at least 12 consecutive months. Inclusion criteria were postmenopausal women aged 48 years or older without hormone replacement therapy. Exclusion criteria were individuals with activity limitations, chronic diseases such as secondary osteoporosis, inflammatory arthritis, metabolic bone disease, malignancy, malabsorption syndromes, hyperthyroidism, hepatic failure, renal failure, and those taking medications that may interfere with bone metabolism, such as glucocorticoids, immunosuppressants, anticonvulsant medications, calcium and vitamin D supplements.

The cut-off points for overweight and obesity recommended by Cooperative Meta-analysis Group of China Obesity Task Force was verified in the large sample survey conducted more recently (13). Based on the results of the survey from the China Kadoorie Biobank Collaborative Group, all participants were classified into normal weight (18.5 ≤ BMI < 24 kg/m^2^), overweight (24 ≤ BMI < 28 kg/m^2^), and obesity (BMI ≥ 28 kg/m^2^) categories. The study was approved by the Ethics Committee of the Third Hospital of Hebei Medical University (Ethics Approval Number: Ke2023-080-1and ke2021-045-1). The research was conducted in accordance with the guidelines of the Declaration of Helsinki and all subjects provided written informed consent.

The validation set comprised data from the National Health and Nutrition Examination Survey (NHANES). This study utilized data from the 2005-2006, 2013-2014, and 2017–2018 cycles. The analysis focused on postmenopausal women aged 48 years or older. Exclusion criteria included male sex, age under 48 years or premenopausal status, pregnancy, and missing body composition data, yielding a final sample of 865 individuals. Participants were categorized according to World Health Organization (WHO) criteria into normal-weight (18.5 ≤ BMI < 25 kg/m²), overweight (25 ≤ BMI < 30 kg/m²), and obese (BMI ≥ 30 kg/m²) groups. The NHANES protocol was approved by the NCHS Research Ethics Review Board, and all participants provided written informed consent. Detailed study design and data are publicly available at https://www.cdc.gov/nchs/nhanes/.

### Data collection

Age (in years) was collected from subjects through self-reported questionnaires and standardized interviews. Participants were considered postmenopausal when they reported having experienced amenorrhea for 12 consecutive months (14). Standard approaches were used to gather anthropometric data. BMI was determined as follow: body weight (kg)/height^2^ (m^2^).

### Body compositions and BMD measurement

Total fat mass (TFM), soft lean mass (SLM), abdominal fat percentage (AF%), hip fat percentage (GF%), AOI, appendicular skeletal muscle mass (ASM), and the BMD values in the lumbar spine (LS), total hip (TH) and femoral neck (FN) were assessed via whole-body DXA scanning (Software Version enCORE 16; Lunar Prodigy, GE Healthcare, USA). Total body fat percentage (TBF%), soft lean mass percentage (SLM%), relative skeletal muscle index (RSMI) were calculated as follows: TBF% was estimated by dividing body fat mass by body weight; SLM% was estimated by dividing body lean mass by body weight; RSMI was calculated as ASM divided by height squared.

### Statistical analysis

Normality of distributions was assessed using the Shapiro-Wilk test. Normally distributed measures were expressed as mean ± standard deviations (SD), and comparisons between BMI subgroups were performed using one-way ANOVA with *post-hoc* LSD tests. Non-normally distributed measures were expressed as median and interquartile range (IQR), and the Kruskal-Wallis test was employed for between-group comparisons. Categorical data are presented as n(%), and group differences were compared using the chi-square test. Spearman correlation analyses were used to explore relationships among different study variables, while multivariate linear regression models were utilized to evaluate relationships among BMD and TFM, AOI, SLM, RSMI, with age serving as fixed covariate. In Model 1, the relationships between TFM, AOI and SLM with BMD in the LS, TH and FN were assessed. Model 2 additionally explored the relationships between RSMI and regional BMD in a model incorporating FM and AOI. The collected data served as the training set for model development, while datasets from the NHANES database were utilized as a validation set to assess model performance. The accuracy of the model was subsequently evaluated by means of multiple linear regression analysis. The results of these analyses were presented as standardized regression coefficients. A P-value of <0.05 was considered significant, and data were analyzed using SPSS v26 (IBM SPSS Statistics) and R (v4.3.1; R Foundation for Statistical Computing).

## Results

### Descriptive statistics

A total of 356 postmenopausal women were included in this study. Their demographic characteristics, anthropometric parameters, body composition components and regional BMD values are compiled in **Table 1**. The mean age of the participants was 62.52 years (range: 48–88 years), and the mean BMI was 24.57 kg/m^2^ (range: 16.03 -35.20 kg/m^2^). The mean TFM was 23.92 kg, representing 38.77% of total body weight, while the mean SLM was 36.71 kg, equating to 59.22% of total body weight. The mean LS BMD was 1.00 g/cm², the mean TH BMD was 0.84 g/cm², and the mean FN BMD was 0.78 g/cm².

**Table 1:** Baseline characteristics of subjects.

Variables	Subjects (n=292)
Age (years)	62.55 ± 7.54
BMI (kg/m^2)^	24.57 ± 3.39
TFM (kg)	23.92 ± 6.08
TBF%	38.77 ± 5.24
AF%	43.90 (38.92,48.85)
GF%	38.25 ± 5.14
AOI	1.13 ± 0.19
SLM (kg)	36.71 ± 4.52
SLM%	58.60 (56.00,61.87)
ASM (kg)	15.68 ± 2.21
RSMI (kg/m^2^)	6.12 ± 0.73
Bone mineral density (g/cm^2^)	
Lumbar spine	1.00 ± 0.17
Hip	0.84 ± 0.14
Femoral neck	0.78 ± 0.13

Values were presented as mean ± standard deviation (SD), median and interquartile range (25^th^,75th). BMI, body mass index; TFM, total fat mass; TBF% was estimated by dividing body fat mass by body weight; SLM, soft lean mass; SLM% was estimated by dividing body lean mass by body weight; AF%, abdominal fat percentage; GF%, hip fat percentage; AOI, android-to-gynoid fat ratio; ASM, appendicular skeletal muscle mass; RSMI was calculated as ASM divided by height squared.

### The relationships among anthropometric, body composition and BMD parameters

Correlations between anthropometrics parameters, body composition components and BMD measurements in different regions are compiled in **Table 2**. These analyses revealed that age was negatively correlated with BMD in all sites (r = -0.354 to -0.254, all P < 0.001). In contrast, higher BMI and TFM were associated with increased BMD in each site (r = 0.181-0.259 and 0.190- 0.255, all *P* < 0.01). Furthermore, SLM, ASM, RSMI were positively associated with BMD in each site (r = 0.339- 0.388, 0.335 - 0.360 and 0.229 - 0.257, all P < 0.01). AOI was positively correlated with BMD in the LS (r = 0.184, *P* < 0.001), while SLM% was inversely correlated with hip BMD (r = -0.109, *P* < 0.05).

**Table 2:** Correlations between anthropometric parameters, body composition components and BMD measurements in different sites.

Variables	LS BMD	TH BMD	FN BMD
Age (years)	-0.254***	-0.335***	-0.354***
BMI (kg/m^2^)	0.231***	0.259***	0.181***
TFM (kg)	0.212***	0.255***	0.190***
TBF%	0.050	0.074	0.034
AOI	0.184***	0.100*	0.099
SLM (kg)	0.352***	0.388***	0.339***
SLM%	-0.085	-0.109*	-0.059
ASM (kg)	0.335***	0.360***	0.357***
RSMI (kg/m^2^)	0.234***	0.257***	0.229**

LS, Lumbar spine; TH, total hip; FN, femoral neck; BMD, bone mineral density; BMI, body mass index; TFM, total fat mass; TBF% was estimated by dividing body fat mass by body weight; SLM, soft lean mass; SLM% was estimated by dividing body lean mass by body weight; AOI, android-to-gynoid fat ratio; ASM, appendicular skeletal muscle mass; RSMI was calculated as ASM divided by height squared. *P<0.05, **P<0.01, ***P<0.001.

As illustrated in **Table 3**, all subjects were categorized into three groups according to BMI values: the normal weight group (n = 157), the overweight group (n = 144) and the obesity group (n = 55). There were no statistically significant differences in age between the three groups. Significant differences were found between the groups in TFM, TBF%, GF%, SLM, ASM and RSMI (all P < 0.05). The following variables demonstrated statistically significant increases with increasing BMI: TFM, TBF%, GF%, SLM, ASM, and RSMI (P for trend < 0.05), with the highest values observed in the obesity group. SLM% decreased with increasing BMI, with the lowest value observed in the obesity group. Compared with the normal weight subjects, the obese and overweight subjects exhibited significantly elevated AF% values (all P < 0.05).

**Table 3:** Comparison of body composition and BMD measurements in different BMI participants.

Variables	Normal weight (n=157)	Overweight (n=144)	Obesity (n=55)	Statistical values (F/H/X^2^)	*P*
Age (years)	62.76 ± 7.86	62.84 ± 7.21	61.20 ± 7.43	1.05	0.350
BMI (kg/m^2^)	21.62 ± 1.72	25.64 ± 1.16	30.17 ± 1.81	689.87	<0.001
TFM (kg)	19.56 ± 4.24	25.44 ± 3.66	32.35 ± 4.72	214.83	<0.001
TBF%	35.87 ± 5.39	40.46 ± 3.68	42.64 ± 3.65	62.92	<0.001
AF%	41.20[34.90,44.65]	46.95[42.03,50.68]	46.20[41.90,51.00]	61.01^#^	<0.001
GF%	36.32 ± 5.21	39.28 ± 4.63	41.05 ± 4.13	25.06	<0.001
AOI	1.08 ± 0.21	1.20 ± 0.16	1.13 ± 0.18	15.51	<0.001
SLM (kg)	34.33 ± 3.35	37.14 ± 3.49	42.40 ± 4.44	104.45	<0.001
SLM%	61.00[58.55,64.05]	57.45[55.16,60.08]	55.15[53.49,57.43]	100.61^#^	<0.001
ASM (kg)	14.57 ± 1.77	15.98 ± 1.77	18.10 ± 2.27	76.95	<0.001
RSMI (kg/m^2^)	5.65 ± 0.53	6.29 ± 0.51	7.05 ± 0.62	147.80	<0.001
Bone mineral density (g/cm^2^)					
Lumbar spine	0.97 ± 0.16	1.00 ± 0.16	1.07 ± 0.19	6.64	0.001
Hip	0.81 ± 0.14	0.85 ± 0.12	0.91 ± 0.18	12.40	<0.001
Femoral neck	0.77 ± 0.14	0.78 ± 0.11	0.83 ± 0.16	5.241	0.006
Osteoporosis [n(%)]	60(38.22)	38(26.39)	11 (20)	8.398^&^	0.015

BMI, body mass index; TFM, total fat mass; TBF% was estimated by dividing body fat mass by body weight; SLM, soft lean mass; SLM% was estimated by dividing body lean mass by body weight; AF%, abdominal fat percentage; GF%, hip fat percentage; AOI, android-to-gynoid fat ratio; ASM, appendicular skeletal muscle mass; RSMI was calculated as ASM divided by height squared. #represents the H-value, ^&^represents the X^2^-value, the remainder test statistic is the F-value.

**Figure 1:**
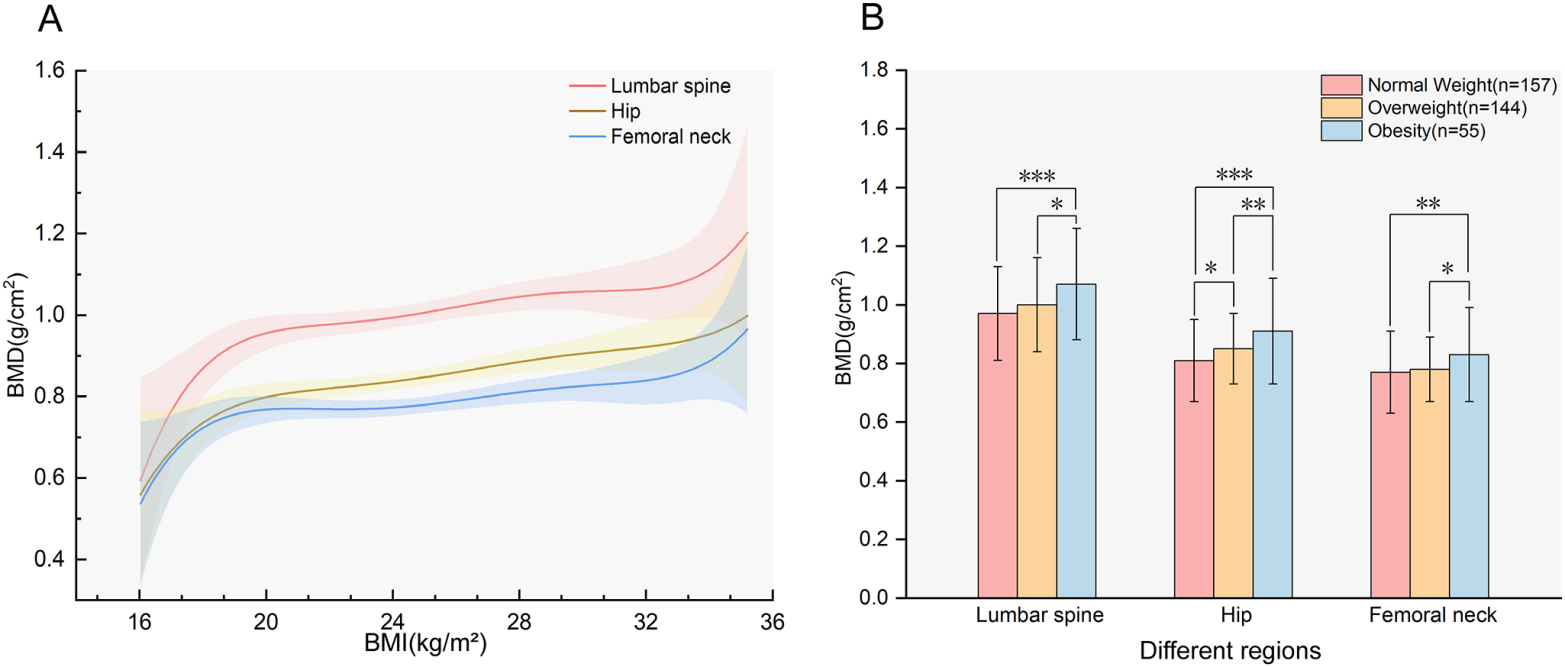
The association between BMI and BMD. **(A)** Smooth fitting curves of BMD with BMI in different regions (P for trend<0.05);**(B)** BMD of participants with different BMI in different regions, the values in the figure represent the mean ± standard deviation. BMD, bone mineral density; BMI, body mass index. *P<0.05; **P<0.01; ***P<0.001.

As illustrated in **Figure 1A**, BMD in all sites increased with increasing BMI. **Figure 1B** indicates the differences in BMD in the LS, TH, and FN among subjects with different BMI values. The results revealed that hip BMD demonstrated a significant increasing trend across ascending BMI categories (P for trend < 0.05). For LS and FN BMD, *post-hoc* comparisons indicated that the obese group had significantly higher BMD than both the normal-weight and overweight groups (P < 0.05), while no other inter-group differences reached statistical significance (all P > 0.05). The prevalence of osteoporosis in the normal-weight, overweight, and obese groups was 38.22%, 26.39%, and 20%, respectively, with the normal-weight group exhibiting the highest prevalence.

### Correlation of body composition parameters and BMD in different BMI participants

**Figure 2:**
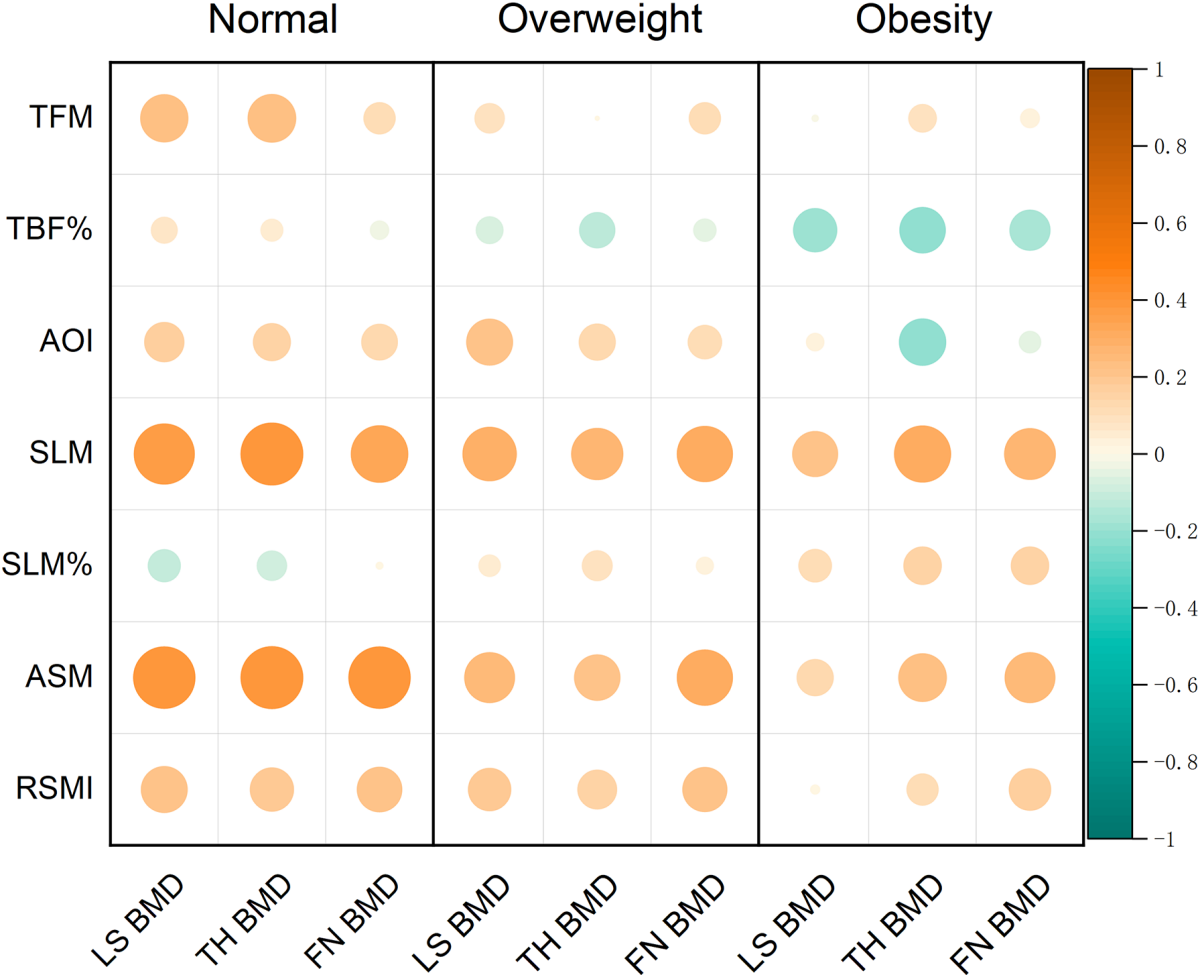
Correlation bubble chart of body composition parameters and BMD measurements of different regions.

Correlations between body composition parameters and BMD in participants with different BMI categories are compiled in **Table 4** and **Figure 2**. **Figure 2** displayed the Spearman correlation bubble chart of body compositions and BMD in different BMI subgroups. Bubble diameters were proportionally scaled to the absolute values of correlation coefficients. A diverging colormap was applied to encode both the magnitude and direction of correlations, spanning continuously from -1 to 1. Specifically, warm orange hues indicate positive correlations while cool blue tones represent negative correlations, with chromatic intensity corresponding to association strength. This dual visual encoding simultaneously conveys effect size through bubble area and correlation polarity through directional hue.

**Table 4:** Correlations between body composition parameters and BMD measurements of different regions in subjects with different BMI.

Variables	Normal weight (n=157)			Overweight (n=144)			Obesity (n=55)		
	LS BMD	TH BMD	FN BMD	LS BMD	TH BMD	FN BMD	LS BMD	TH BMD	FNBMD
Age (years)	-0.415***	-0.460***	-0.483***	-0.110	-0.206*	-0.243**	-0.119	-0.306*	-0.202
TFM (kg)	0.226**	0.233**	0.104	0.091	0.003	0.101	-0.005	0.082	0.038
TBF%	0.072	0.052	-0.036	-0.077	-0.128	-0.054	-0.191	-0.212	-0.166
AOI	0.209**	0.146	0.129	0.192*	0.131	0.114	0.046	-0.268*	-0.140
SLM (kg)	0.364***	0.388***	0.326***	0.291***	0.266**	0.314***	0.206	0.319*	0.262
SLM%	-0.107	-0.092	0.006	0.049	0.095	0.032	0.110	0.145	0.144
ASM (kg)	0.383***	0.386***	0.383***	0.254**	0.212*	0.310***	0.136	0.233	0.256
RSMI (kg/m^2^)	0.214**	0.194*	0.203*	0.186*	0.154	0.201*	0.010	0.104	0.176

LS, Lumbar spine; TH, total hip; FN, femoral neck; BMD, bone mineral density; TFM, total fat mass; TBF% was estimated by dividing body fat mass by body weight; SLM, soft lean mass; SLM% was estimated by dividing body lean mass by body weight; AOI, android-to-gynoid fat ratio; ASM, appendicular skeletal muscle mass; RSMI was calculated as ASM divided by height squared. *P < 0.05, **P < 0.01, ***P < 0.001.

In the normal weight subjects, age was negatively correlated with BMD in the all sites (r = -0.483 – -0.415, all *P* < 0.001). Conversely, TFM was positively correlated with LS and TH BMD (r = 0.226 and 0.233, both *P* < 0.05). Similarly, AOI was positively correlated with LS BMD (r = 0.209, *P* < 0.05). Furthermore, SLM, ASM and RSMI were positively correlated with BMD in the all sites (r = 0.326 – 0.388, r = 0.383 – 0.386 and r = 0.194 – 0.214, all *P* < 0.05).

In the overweight subjects, age was negatively correlated with the TH and FN BMD (r = -0.206 and -0.243, *P* < 0.05). However, SLM and ASM were found to be positively correlated with BMD in the all sites (r =0.266 – 0.314 and r = 0.212 – 0.310, all *P* < 0.05), and RSMI was positively correlated with the LS and the FN BMD (r = 0.186 and 0.201, all *P* < 0.05).

In the obese subjects, both age and AOI exhibited significant inverse correlations with TH BMD (r = -0.306, -0.268, *P* < 0.05). In contrast, SLM was positively correlated with TH BMD (r = 0.319, *P* < 0.05). However, no significant correlations were observed between TFM, ASM, RSMI and BMD in any site.

### Multivariate analyses in different BMI participants

Multivariate linear regression analyses were performed to evaluate the associations between different variables and BMD (**Table 5**). Regression model 1 was established with LS, TH and FN BMD as dependent variables respectively, and TFM, AOI and SLM as independent variables. Regression model 2 was established with LS, TH and FN BMD as dependent variables respectively, and TFM, AOI and RSMI as independent variables. The age of the subjects was treated as a fixed covariate. Hypothesis tests for equality of multiple covariance matrices were performed in these models, which revealed the absence of multicollinearity among the independent variables (all of VIF values close to 1).

**Table 5:** Multivariate linear regression analysis of TFM、AOI、SLM or RSMI and BMD of different regions in participants with different BMI.

Variables	LS BMD (g/cm^2^)				TH BMD (g/cm^2^)				FN BMD (g/cm^2^)			
	Sβ	*t*	*P*	VIF	Sβ	*t*	*P*	VIF	Sβ	*t*	*P*	VIF
Normal weight (n=157) Model 1												
TFM(kg)	0.030	0.376	0.707	1.529	0.038	0.466	0.642	1.529	-0.108	-1.296	0.197	1.529
AOI	**0.289**	**3.697**	**<0.001**	1.430	**0.224**	**2.852**	**0.005**	1.430	**0.274**	**3.383**	**0.001**	1.430
SLM(kg)	**0.313**	**4.503**	**<0.001**	1.129	**0.284**	**4.077**	**<0.001**	1.129	**0.260**	**3.613**	**<0.001**	1.129
Model 2												
TFM(kg)	0.143	1.797	0.074	1.397	0.140	1.775	0.078	1.397	-0.015	-0.183	0.855	1.397
AOI	**0.235**	**2.94**	**0.004**	1.403	**0.174**	**2.192**	**0.030**	1.403	**0.228**	**2.796**	**0.006**	1.403
RSMI(kg/m^2^)	**0.218**	**3.229**	**0.002**	1.003	**0.211**	**3.147**	**0.002**	1.003	**0.182**	**2.630**	**0.009**	1.003
Overweight (n=144) Model 1												
TFM(kg)	0.028	0.348	0.728	1.036	-0.037	-0.449	0.654	1.036	0.054	0.672	0.503	1.036
AOI	**0.207**	**2.527**	**0.013**	1.037	0.142	1.741	0.084	1.037	0.131	1.633	0.105	1.037
SLM(kg)	**0.238**	**2.895**	**0.004**	1.050	**0.245**	**2.982**	**0.003**	1.050	**0.246**	**3.044**	**0.003**	1.050
Model 2												
TFM(kg)	0.065	0.790	0.431	1.006	-0.001	-0.007	0.994	1.006	0.088	1.094	0.276	1.006
AOI	**0.193**	**2.239**	**0.027**	1.095	0.119	1.388	0.167	1.095	0.095	1.136	0.258	1.095
RSMI(kg/m^2^)	0.100	1.186	0.238	1.061	0.141	1.673	0.097	1.061	**0.196**	**2.380**	**0.019**	1.061
Obesity (n=55) Model 1												
TFM(kg)	-0.056	-0.378	0.707	1.184	0.021	0.144	0.886	1.184	0.019	0.130	0.897	1.184
AOI	0.225	1.275	0.208	1.700	-0.132	-0.745	0.460	1.700	-0.077	-0.436	0.665	1.700
SLM(kg)	0.309	1.822	0.074	1.574	0.049	0.286	0.776	1.574	0.115	0.672	0.505	1.574
Model 2												
TFM(kg)	0.010	0.067	0.947	1.114	0.034	0.239	0.812	1.114	0.044	0.306	0.761	1.114
AOI	0.084	0.506	0.615	1.424	-0.189	-1.183	0.242	1.424	-0.138	-0.847	0.401	1.424
RSMI(kg/m^2^)	-0.011	-0.081	0.936	1.037	-0.179	-1.319	0.193	1.037	-0.048	-0.342	0.734	1.037

All models were adjusted for age in postmenopausal women. All significant values are shown in bold.

Sβ, standardized β; LS, Lumbar spine; TH, total hip; FN, femoral neck; BMD, bone mineral density. TFM, total fat mass; SLM, soft lean mass; AOI, android-to-gynoid fat ratio; VIF, variance inflation factor; ASM, appendicular skeletal muscle mass; RSMI was calculated as ASM divided by height squared.

In the normal weight subjects, model 1 indicated that AOI and SLM were positively associated with BMD in LS (Sβ=0.289; 0.313), TH (Sβ=0.224; 0.284), and FN (Sβ=0.274; 0.260). model 2 confirmed these associations for both AOI (Sβ=0.235, 0.174, 0.228) and RSMI (Sβ=0.218, 0.211, 0.182).

In the overweight subjects, model 1 showed positive associations of AOI with LS BMD (Sβ=0.207) and of SLM with BMD in all three sites (Sβ=0.238, 0.245, 0.246). In model 2, only AOI retained its association with LS BMD (Sβ=0.193), and RSMI was specifically associated with FN BMD (Sβ=0.196).

Finally, in the obese subjects, Neither model 1 nor model 2 revealed any significant associations between the body composition parameters (TFM, AOI, SLM, RSMI) and BMD at any site.

### External validation with NHANES database

For external validation, data from the NHANES cycles (2005-2006, 2013-2014, 2017-2018) were compiled. After applying the inclusion and exclusion criteria, 865 individuals constituted the validation cohort, which was subsequently analyzed using multiple linear regression. The beta coefficients (β) with 95% confidence intervals (95% CI) from the training set and the corresponding β from the validation set are summarized in **Table 6**.

**Table 6:** External validation with the NHANES database.

Variables	LS BMD (g/cm^2^)			TH BMD (g/cm^2^)			FN BMD (g/cm^2^)		
	Training set β	95% CI	Validation set β	Training set β	95% CI	Validation set β	Training set β	95% CI	Validation set β
Normal weight (n=157) Model 1									
TFM (kg)	0.001	(-0.005, 0.007)	**-0.006**	0.001	(-0.004, 0.006)	-0.002	-0.004	(-0.009, 0.002)	-0.001
AOI	**0.229**	**(0.106, 0.351)**	0.001	**0.145**	**(0.044, 0.245)**	**0.111**	**0.182**	**(0.076, 0.288)**	0.070
SLM (kg)	**0.015**	**(0.009, 0.022)**	**0.013**	**0.011**	**(0.006, 0.017)**	**0.009**	**0.011**	**(0.005, 0.017)**	**0.008**
Model 2									
TFM (kg)	0.006	(-0.001, 0.012)	-0.001	0.004	(-0.001, 0.009)	0.002	-0.487×10^-3^	(-0.006, 0.005)	0.002
AOI	**0.185**	**(0.061, 0.31)**	-0.044	**0.112**	**(0.011, 0.214)**	**0.079**	**0.151**	**(0.044, 0.258)**	0.041
RSMI (kg/m^2^)	**0.068**	**(0.026, 0.109)**	**0.060**	**0.054**	**(0.02, 0.087)**	**0.048**	**0.047**	**(0.012, 0.083)**	**0.037**
Overweight (n=144) Model 1									
TFM (kg)	0.001	(-0.006, 0.008)	-0.001	-0.001	(-0.007, 0.004)	-0.003	0.002	(-0.003, 0.006)	**-0.004**
AOI	**0.208**	**(0.045, 0.37)**	-0.031	0.107	(-0.015, 0.23)	**0.114**	0.092	(-0.019, 0.203)	0.047
SLM (kg)	**0.011**	**(0.003, 0.018)**	**0.012**	**0.008**	**(0.003, 0.014)**	**0.008**	**0.008**	**(0.003, 0.013)**	**0.007**
Model 2									
TFM (kg)	0.003	(-0.0041, 0.01)	0.005	-0.199×10^-4^	(-0.005, 0.005)	0.165×10^-3^	0.003	(-0.002, 0.007)	-0.001
AOI	**0.194**	**(0.023, 0.365)**	-0.049	0.09	(-0.038, 0.218)	0.100	0.067	(-0.049, 0.182)	0.033
RSMI (kg/m^2^)	0.031	(-0.021, 0.084)	**0.035**	0.033	(-0.006, 0.072)	**0.031**	**0.043**	**(0.007, 0.078)**	**0.030**
Obesity (n=55) Model 1									
TFM (kg)	-0.002	(-0.014, 0.01)	-0.001	0.001	(-0.01, 0.012)	0.129×10^-3^	0.001	(-0.009, 0.011)	0.135×10^-3^
AOI	0.243	(-0.14, 0.626)	0.021	-0.129	(-0.478, 0.219)	**0.122**	-0.068	(-0.379, 0.244)	0.002
SLM (kg)	0.014	(-0.001, 0.028)	**0.007**	0.002	(-0.012, 0.016)	**0.005**	0.004	(-0.008, 0.016)	**0.006**
Model 2									
TFM (kg)	0.408×10^-3^	(-0.012, 0.013)	0.002	0.001	(-0.009, 0.012)	0.001	0.001	(-0.008, 0.011)	0.002
AOI	0.091	(-0.271, 0.453)	0.052	-0.185	(-0.499, 0.129)	**0.144**	-0.121	(-0.407, 0.166)	0.031
RSMI (kg/m^2^)	-0.004	(-0.094, 0.086)	0.005	-0.051	(-0.129, 0.027)	**0.024**	-0.012	(-0.083, 0.059)	**0.028**

All models were adjusted for age in postmenopausal women. All significant values are shown in bold.

LS, Lumbar spine; TH, total hip; FN, femoral neck; BMD, bone mineral density. TFM, total fat mass; SLM, soft lean mass; AOI, android-to-gynoid fat ratio; ASM, appendicular skeletal muscle mass; RSMI was calculated as ASM divided by height squared; 95% CI, 95% Confidence Interval.

In the normal weight group, validation of model 1 confirmed positive associations of SLM with LS (β=0.013), TH(β=0.009), and FN(β=0.008; all *P* < 0.05) BMD and of AOI with TH BMD(β=0.111, P < 0.05); all β coefficients were within the training set’s 95% CIs. model 2 validation similarly showed positive associations for RSMI with BMD at all three sites(β=0.06, 0.048, 0.037; all P < 0.05) and for AOI with hip BMD(β=0.079, P < 0.05), with all β values contained within the training set’s CIs.

In the overweight group, model 1 validation confirmed SLM’s positive association with BMD at all sites (β=0.012, 0.008, 0.007, respectively; all *P* < 0.05), and Model 2 confirmed RSMI’s association with femoral neck BMD(β=0.030, *P* < 0.05), all consistent with the training set’s CIs.

In the obese group, the validation set indicated positive associations for SLM and AOI with BMD in both models. However, in stark contrast, the original training set for the obese group revealed no significant associations for any body composition parameters with BMD in either model.

## Discussion

In this study, postmenopausal female were selected for body compositions and BMD measurements. The correlations between body composition components and BMD were observed in subjects with normal weight, overweight and obesity. It was demonstrated that BMI was correlated with BMD in postmenopausal women. Meanwhile, BMD in all sites exhibited a tendency to increase with increasing BMI. In addition, the body composition parameters TFM, SLM, ASMI, RSMI were found to be correlated with BMD in all sites. The correlations between body compositions and BMD in each site were found to vary according to changes of BMI categories. Likewise, there were site-specific differences in the correlation between body compositions and BMD in postmenopausal women. The robustness of these findings was confirmed through external validation in an independent cohort from the NHANES database.

Postmenopausal women represent the most prevalent demographic with OP. A study about the 2018 China Osteoporosis Epidemiological Survey indicated that the prevalence of OP in postmenopausal women aged 40 years and above is 32.5%. Furthermore, the prevalence of OP according to the classification of body weight as low weight, normal weight, overweight, and obesity is 69.9%, 42.2%, 24.2%, and 14.6%, respectively (15). This suggests that increased body weight may act as a protective factor for OP. Currently, BMD measurement and assessment is the primary method for diagnosing OP. Previous studies have demonstrated correlations between BMI and BMD in the lumbar spine and hip in postmenopausal women, with higher BMI being associated with higher BMD (16). The results of our study were consistent with those of previous studies, indicating that an increase in BMI was associated with a trend towards increased lumbar spine, femoral neck, and hip BMD. It is currently believed that the mechanism of increased BMD in obese patients can be attributed, on the one hand, to increased mechanical loads and stresses due to elevated body weight (17). On the other hand, it may be associated with metabolic alterations resulting from changes in body composition (18).

Body weight is mainly composed of lean body mass (LBM or LM) and fat mass. DXA is part of the three-compartment model, which divides the body into fat, bone mineral and all other non-bone lean tissue. The term ‘SLM’ encompasses total water, total protein and extraosseous inorganic salts. Similarly, the term ‘LM’ encompasses SLM and intraosseous inorganic salts. BMI cannot distinguish body composition components, LM and FM have different roles in bone health. Systematic reviews have shown that both LM and FM are associated with BMD (19, 20). A study of an adult population cohort identified a strong positive association between ASM and BMD in both men and women (21). Moreover, in the cohort of non-obese postmenopausal women over the age of 60 from China, FM was positively associated with BMD, while AOI was negatively correlated with BMD (9). The results of our study indicated that TFM, SLM and ASM were positively correlated with BMD in all sites in postmenopausal women. These findings are consistent with previous results. However, our study found that AOI was positively correlated with BMD in the lumbar spine, which differs from above result. Nevertheless, a study from the Czech Republic indicated a positive correlation between AOI and lumbar BMD in postmenopausal women (22). A two-sample Mendelian randomization analysis indicated that there was a causal and positive association between waist-to-hip ratio and BMD (23). Thus, the results of studies on the correlation between fat distribution and BMD are highly heterogeneous. Further studies have demonstrated that the correlation between abdominal obesity and BMD may vary according to age, sex, and BMI (24, 25).

Hence, the present study was conducted to investigate the correlation between body composition components and BMD in postmenopausal women according to BMI classifications. The study yielded an intriguing result, the correlations between body composition components and BMD exhibited BMI classification-specific variations in different sites. In normal body weight participants, the inclusion of TFM, AOI and SLM in the regression model resulted in the following correlations: SLM and AOI were positively associated with BMD at all measured sites (lumbar spine, hip, and femoral neck). Crucially, this relationship held true when the model was adjusted using RSMI instead of SLM. These consistent results point to a beneficial role of both increased lean mass and a central fat distribution on BMD in this specific demographic. Consistent with normal-weight findings, the overweight group showed positive correlations of SLM, ASM, and RSMI with all-site BMD, and of AOI with lumbar spine BMD. In adjusted models, only SLM and AOI remained significant, the latter specifically for the lumbar spine, suggesting a site-specific benefit of abdominal fat. Among obese individuals, univariate correlations of AOI and SLM with hip BMD vanished after multivariate adjustment. This confirms the BMI-dependent and site-specific nature of these relationships. While these relationships are most evident in normal/overweight women, interventions targeting abdominal fat reduction and muscle mass increase may support BMD in obesity.

External validation using the NHANES cohort confirmed the beneficial associations of SLM and AOI with BMD across multiple skeletal sites in normal-weight and overweight postmenopausal women, meeting the pre-specified validation criterion defined as the validation set’s β coefficients falling within the 95% CIs of the training set. A notable exception was a negative association between TFM and lumbar spine BMD specific to the normal weight validation set, a finding potentially explained by population heterogeneity in adiposity and the broader BMI distribution of the external cohort. Whereas the validation set indicated positive associations for SLM and AOI with BMD in the obese group, the training set showed no such associations. A likely explanation for this discrepancy is the limited statistical power in the training set, attributable to the relatively small sample size of the obese subgroup.

The precise mechanism by which FM affects bone mass remains unclear. It is possible that, due to the fact that both adipocytes and osteoblasts originate from MSCs (26), the competition between adipogenic and osteogenic differentiation may result in a reduction in osteogenesis when there is an increase in adipogenesis. What’s more, adipose tissue, which is a primary source of aromatase, has been demonstrated to favor estrogen synthesis, thereby promoting bone formation and reducing bone resorption effects (27). Consequently, the impact of FM on BMD may be contingent upon the ultimate consequence of the interaction between these mechanisms.

Most studies on the correlation between FM and BMD have not been categorized according to BMI. A study in a group of normal-weight middle-aged Koreans (28) demonstrated that an increase in percentage body fat was associated with a decrease in BMD. Analysis of the reasons for the discrepancy in results may be attributed to differences in study methodology. The model employed in the aforementioned study only adjusted for age, BMI, and lifestyle, and did not correct for other body components. Our study did not consider the effect of lifestyle.

In addition, the result of our study indicated a positive correlation between AOI and lumbar spine BMD in normal weight (BMI range: 21.62 ± 1.72 kg/m²) postmenopausal women. In contrast, another study identified a significant negative correlation between waist-to-hip ratio and lumbar spine BMD (BMI range: 27.70 ± 4.73 kg/m^2^) (29). In addition to BMI differences, another study did not consider the effects of other body components. The discrepancies observed may be attributed to the disparate age profiles of the subjects (69.12 ± 5.17 *vs* 63.08 ± 8.03) and different statistical analysis. Furthermore, there are studies that corroborate our findings. For instance, there is a positive correlation between AOI and lumbar spine BMD in the Thai female population over 40 years of age (BMI range: 23.8 ± 3.8 kg/m^2^) (12). A recently published cross-sectional study demonstrated a positive correlation between AOI and lumbar spine BMD in postmenopausal women (BMI range: 26.1 ± 4.0 kg/m²). This positive correlation has been attributed to biomechanical effects, with higher AOI increasing the loads on the medial bones, particularly the spine. This, in turn, leads to an increase in BMD at the corresponding sites (22).

According to Wolff’s Law, bone adapts its structure to withstand prevailing mechanical forces. In normal weight women, a high AOI reflects a pattern of central adiposity that imposes a greater gravitational load on the axial skeleton. This, in turn, may elicit an anabolic skeletal adaptation, whereby weight-bearing bones like the lumbar spine increase their BMD for structural reinforcement. The underlying biomechanical hypothesis is that the fat mass provides a direct osteogenic stimulus, activating bone-forming osteoblasts. Supporting the complexity of this relationship, a study in US adults (30), while primarily reporting an inverse correlation between the metabolic score for visceral fat (METS-VF) and lumbar spine BMD, also highlighted a significant nonlinear association between these variables, with a significant inflection point at METS-VF=5.47. Suggesting that visceral adipose metabolic load negatively affects BMD after reaching a certain level. This phenomenon may be related to the protective effect of mechanical loading or fat-derived estrogens on bone tissue when fat levels are low. Beyond mechanical loading, moderate abdominal fat accumulation in normal-weight, metabolically healthy women may be accompanied by a physiological increase in leptin. This hormone can indirectly stimulate bone formation by acting on the hypothalamus to promote sympathetic nervous activity (31). Furthermore, leptin may also exert a direct effect by binding to receptors on osteoblasts, thereby promoting their differentiation and activity.

Previously studies have suggested that the most important body component influencing BMD in postmenopausal women is LM, which is significantly associated with all BMD sites (32). Nevertheless, a cross-sectional study of postmenopausal women with a BMI of 26.1 ± 4.0 kg/m^2^ demonstrated that there was no correlation between LM and BMD in any site (22). Our study identified that SLM was positively correlated with BMD at the lumbar spine, hip, and femoral neck in both the normal weight (BMI 21.62 ± 1.72 kg/m²) and overweight (BMI 25.64 ± 1.16 kg/m²) groups. Interestingly, no correlation was observed between SLM and BMD in any site in obese women (BMI range:30.17 ± 1.81 kg/m^2^). A study of postmenopausal women with a BMI of 22.31 ± 2.91 kg/m^2^ also indicated positive correlations between LM and BMD at the lumbar spine, hip, and femoral neck (33), in line with our findings. Other than that, it has been proposed that assessing ASM may be a more suitable approach for evaluating muscle mass than LM (34). A study (n=114) demonstrated that there was no correlation between RSMI and BMD in postmenopausal women (35). Nevertheless, another study comprising 948 subjects (465 women) aged 40 to 59 years (BMI range: 28.9 ± 7.1 kg/m²) demonstrated a positive correlation between RSMI and lumbar spine BMD (36). Our findings demonstrate that RSMI was positively associated with BMD at all sites in normal weight postmenopausal women, whereas a positive association was observed only with femoral neck BMD in the overweight subjects. The apparent contradiction of these results suggests that the impact of SLM on BMD is intricate and is not solely influenced by factors such as age, BMI, ethnicity, and lifestyle. In addition, it is also related to the methodology of the study, which requires further research to provide more robust evidence.

There are multiple strengths to the present study. Firstly, we ascertained the influence of body composition factors, including TFM, AOI, SLM, ASM and RSMI, on lumbar spine, hip and femoral neck BMD in postmenopausal women. Secondly, our results revealed correlations between body composition and BMD in postmenopausal women with different BMI classifications. These results were further confirmed by external validation with the NHANES database, thereby providing new insights that address a notable gap in the existing literature.

There are a number of limitations to the present study. For one, this study was cross-sectional in design, thus precluding our ability to draw causal inferences pertaining to the relationships between body composition and BMD. Secondly, this study did not include additional factors that influence BMD, such as marital status, reproductive history, and especially age at menopause and parity, occupational status, lifestyle, and vitamin D levels. Additionally, this study did not further explore the relationship between regional BMD and localized body composition, which may have influenced the interpretation of the results. Thirdly, the total number of participants in this study was relatively small, which was particularly evident in the obese group and resulted in an uneven distribution across the BMI strata. This imbalance may constrain the statistical power and precision of our subgroup analyses.

## Conclusions

There is a correlation between body compositions and BMD in postmenopausal women. The impact of body compositions on BMD demonstrated variations in BMI classification and site-specific differences. Increased abdominal fat may confer a potential benefit for BMD in non-obese women with relative metabolic health. Conversely, optimizing body composition by reducing body fat and increasing muscle mass remains crucial for skeletal health in postmenopausal women.

## Data Availability

The raw data supporting the conclusions of this article will be made available by the authors, without undue reservation.
